# MoS_2_/C Multilayer Nanospheres as an Electrode Base for Lithium Power Sources

**DOI:** 10.1186/s11671-016-1451-4

**Published:** 2016-05-04

**Authors:** Lyudmyla O. Shyyko, Volodymyr O. Kotsyubynsky, Ivan M. Budzulyak, Piotr Sagan

**Affiliations:** Vasyl Stefanyk Precarpathian National University, 57 Shevchenko Str., Ivano-Frankivsk, 76018 Ukraine; Center for Innovation and Transfer of Natural Sciences and Engineering Knowledge, University of Rzeszow, 1 Pigonia Str., Rzeszow, 35959 Poland

**Keywords:** MoS_2_, Carbon, Multilayer nanospheres, Electrical conductivity, Specific capacity

## Abstract

Multilayer nanospheres with alternating 2H-MoS_2_ and C layers were studied as a cathode base for lithium power sources. Interesting hierarchical structure, synergetic effect, and the presence of defects as supplementary active sites, introduced by the additional annealing at 773 K in Ar atmosphere, have determined the conductivity, referred to symmetric hopping or random barrier model, and led to achieve the high values of specific capacity of 3700, 1390, and 790 A h kg^−1^ at currents 0.1, 0.3, and 0.5 C. Such unusual result was never reported before and could be explained by combining of the faradaic and non-faradaic accumulation processes within electrode material.

## Background

Since the batteries were introduced in 1980s as new high-energy density power sources, one of the most promising materials used as electrode materials for further commercialization was MoS_2_. Being a layered transition metal dichalcogenide compound, it possesses the ability to accommodate Li ions within interlayer spaces that are due to the noncovalent Van der Waals forces between S-Mo-S packages. Taking into account its low cost, owing to the natural abundance, the first MoS_2_-based commercial batteries were developed by Moli Energy Ltd., (British Columbia, Canada) in 1989 and have the specific energy values 100 W h kg^−1^ [1, 2]. In spite of few failures in operating such systems, the study of MoS_2_ as an electrode material still is continuing.

Theoretically, the capacity of MoS_2_ is about 167 A h kg^−1^, when in conversion reaction, only one mole of Li^+^ takes part per mole of MoS_2_, but reported typical capacity value even for bulk material is more than 600 A h kg^−1^ and became a reason to consider at least four Li^+^ ions intercalated per MoS_2_ unit. According to [3], it takes around six lithium ions during the first discharge cycle. However, the first discharge process capacity of the MoS_2_ quickly decreases by several times. Most of the intercalated Li^+^ ions remain localized in the crystal structure within the interlayer space between S-Mo-S packages after the first discharge in nanostructures despite that the Li^+^ diffusion path is significantly shortened in comparison to bulk material. Both bulk and exfoliated materials exhibit capacity reduction upon cycling; moreover, for exfoliated MoS_2_, this decrease can be more sharp [4, 5]. The initial discharge capacity of exfoliated MoS_2_ is typically more than 1000 A h kg^−1^, and capacity gain is caused by the Li_2_S formation and Mo metal reduction. MoS_2_ can be additionally exfoliated via lithium intercalation with a metastable phase formation: electron transfer from lithium during the intercalation causes the change in the electron density and the additional deformation of crystal structure, in particular, in Mo symmetry—from trigonal prismatic (2H) to octahedral. The increasing of MoS_2_ structural disorder leads to the possibility for more Li^+^ ions to reversibly penetrate into the expanded interlayer spaces. But still, the capacity of exfoliated MoS_2_ dramatically decreases with the increasing of cycle number.

The electrochemical performance of MoS_2_ as an electrode for lithium batteries was believed to be significantly influenced by morphology, structure, and particle size. In order to shorten the Li^+^ diffusion path for improving the performance, many research efforts have been directed to prepare nanostructured MoS_2_ for application as electrode material. The most popular approach to increase the capacity is to enlarge the interlayer distance and lower the barrier for Li^+^ intercalation. A good example is MoS_2_ nanoplates [6], consisting of disordered graphene-like layers, with a thickness of ∼30 nm and interlayer distance of 0.69 nm (for bulk, it is 0.62 nm), that showed reversible capacity of 700 A h kg^−1^ even at 50 C. Another way is to play on morphology effect; 3D MoS_2_ nanospheres [7] and flower-like structures [8] have been demonstrated with the reversible capacity of >850 A h kg^−1^ however at low current rates. In the same time, 1D nanoribbons and nanotubes possess 776 A h kg^−1^ [9, 10].

But the latest tendency is a combination of MoS_2_ with carbon materials (as nanotubes, carbon coating, and graphene). Being a good conductor and chemically stable substance, it contributes to the overall conductivity of the composite, facilitating the charge transfer within the material, and prevents the volume expansion and restacking of MoS_2_. The synergetic effect between these two materials improves the electrochemical performance of the composite in Li batteries. Indeed, a MoS_2_/GNS (graphene nanosheets) composite with a Mo to C mole ratio of 1:2 that delivered the highest specific capacity (1300 A·h·kg^-1^), although the specific capacity of samples with mole ratios of 1:1 and 1:4 (i.e., 1001 A·h·kg^-1^and 1132 A·h·kg^-1^, respectively) still exhibited high specific capacity and better cycling stability than pure MoS_2_ and GNS[11]. One of the largest reported value of specific capacity is 1549 A·h·kg^-1^ for 2D MoS_2_ grown on the surface of 1D multiwall carbon nanotubes [12]. At the same time, it should be taken into account the possibility of pseudocapacitive mechanism of charge storage with electron transfer and oxidation/reduction of Mo^4+^ [13]. In some cases, the contribution of pseudocapacity is dominant and has significant impact on the electrochemical performance [14].

Here, we present hierarchically structured nanospheres with alternating layers of MoS_2_ and carbon as an electrode base for lithium power sources. Studied nanocomposite showed the very high specific capacity during Li^+^ intercalation and interesting conductivity features.

## Methods

**Fig. 1:**
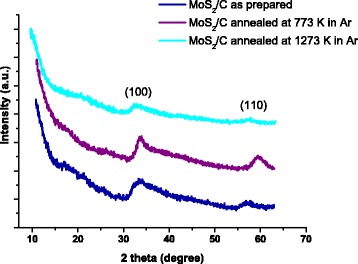
XRD patterns of MoS_2_/C multilayer nanospheres

**Fig. 2:**
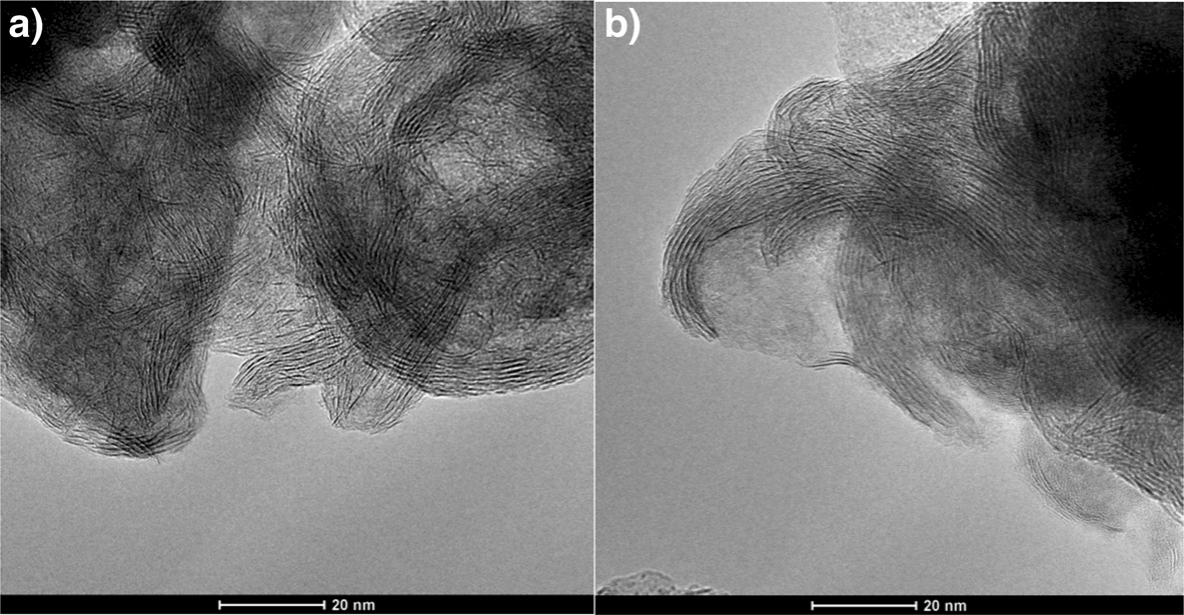
TEM images of as-prepared MoS_2_/C (**a**) and annealed in Ar at 773 K (**b**)

**Fig. 3:**
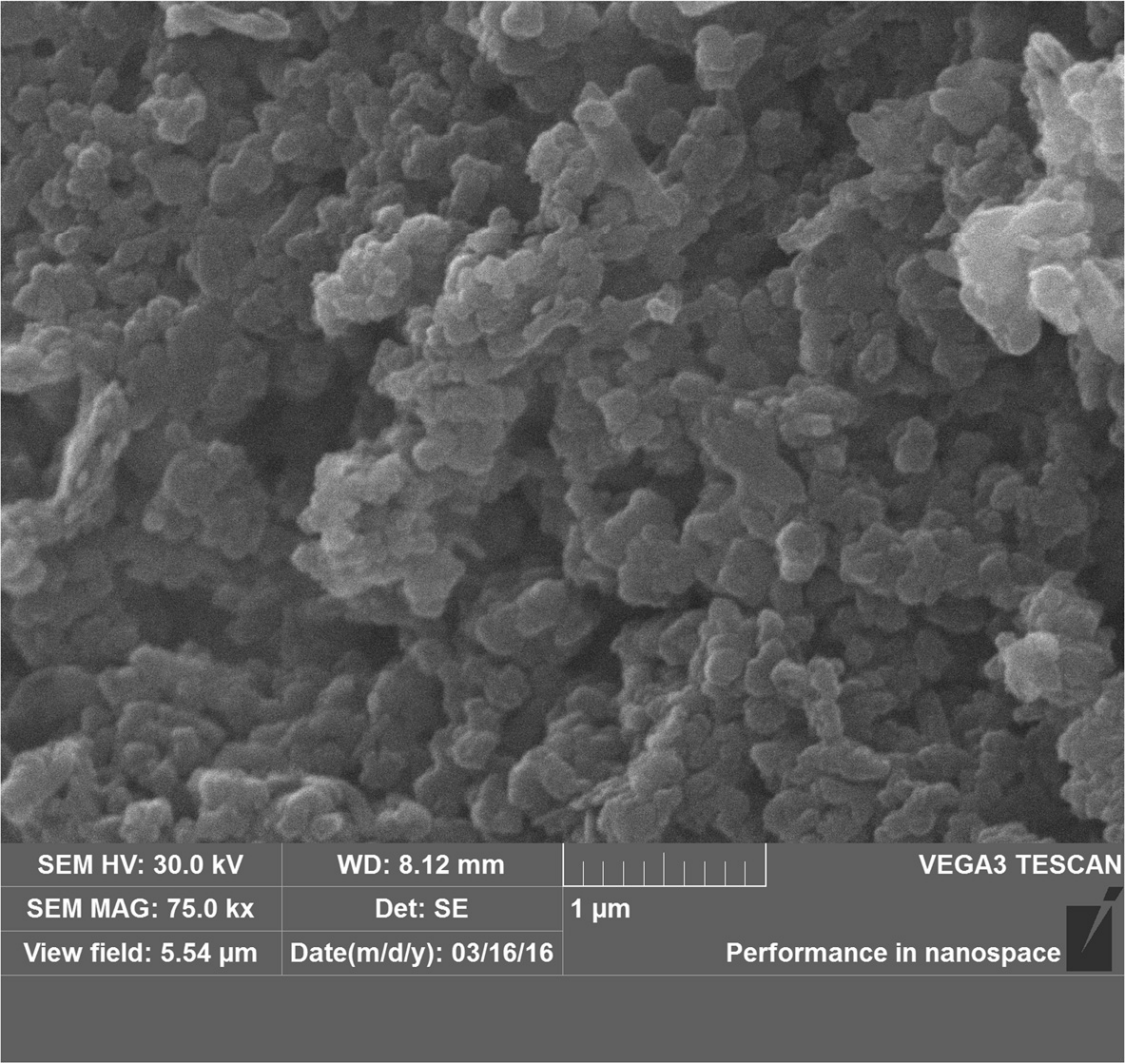
SEM image of MoS_2_/C

The synthesis procedure was based on the hydrothermal method described in [15]. XRD study confirmed the formation of 2H-MoS_2_ (P63/mmc) (Fig. 1); TEM, SEM, and EDS investigations (FEI Technai G2 X-TWIN and VEGA 3 TESCAN microscopes) showed that the obtained nanocomposite consists of mostly spherical particles with a size of near 40–70 nm composed of alternating layers of 2H-MoS_2_ and carbon (Fig. 2a, Fig. 3). Additional annealing led to partial rupture of the spherical particles due to gas releasing during the surfactant decomposition (Fig. 2b), but the relative contents of Mo, S, and C atoms, received from EDS analysis, remain unchanged (Table 1).

**Table 1:** The relative element contents received from EDS

	MoS_2_/C as-prepared, at.%	MoS_2_/C after annealing, at.%
Mo	15.8 ± 0.6	13.0 ± 0.6
S	27.0 ± 0.6	23.0 ± 0.5
C	46.8 ± 0.4	53.9 ± 0.5

Electrical conductivity *σ* as a function of frequency (0.01–100 kHz range) and temperature were measured by the method of impedance spectroscopy (Autolab, PGSTAT12, FRA-2 software). All samples were made in pellet form with the diameter of 1.7 × 10^−2^ m and thickness of 0.6 × 10^−3^ m under pressure of 34 MPa. Taking into account the nature of ultrafine material to avoid, the probable oxidation of the air at it was chosen to conduct the conductivity evaluation is in the narrow temperature range of 293–333 K with precision of ±1 K.

The galvanostatic and potentiodynamic measurements were conducted in two electrode cells with Li as a counter electrode and 1М LiPF_6_ in a 50:50 (*w*/*w*) mixture of ethylene carbonate and diethyl carbonate as an electrolyte. The working electrode consists of a test material (MoS_2_/C multilayered nanospheres), carbon black, and polyvinylidene difluoride (PVDF) in a weight ratio of 8:1:1 coated on Cu foil.

## Results and Discussion

**Fig. 4:**
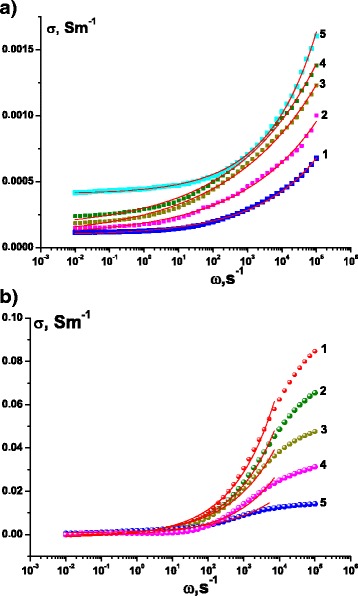
The frequency dependence of the real part of the conductivity of the nanocomposite MoS_2_/C with spherical multilayer particles, obtained at temperatures of 293 (*1*), 303 (*2*), 313 (*3*), 323 (*4*), and 333 K (*5*). **a** As-prepared material. **b** After annealing in argon at 773 K

Frequency dependence of the real part of the conductivity of the MoS_2_/C nanosphere samples dried at 353 K (Fig. 4a) has the typical look of condensed disordered dielectrics and semiconductors: asymptotic approximation to a certain value at constant current and constant power dependence at high frequencies. Meanwhile, as it is seen in Fig. 4b, the frequency dependence of conductivity of MoS_2_/C annealed in Ar atmosphere at 773 K differs. TEM confirmed that heat treatment led to partial destruction of multilayer nanospheres; however, the XRD patterns are similar indicating on the rigidity of 2H-MoS_2_ structure of the material. The morphology changes dramatically affect the electrical properties of the material and the value of conductivity, being increased from 0.4 × 10^−4^ to 1.95 × 10^−4^ Sm^−1^. The typical conductivity of MoS_2_ is in the range of 10^–6^–10^–8^ Sm^–1^ [16, 17], and the achieved conductivity growth in our case should have an influence on the overall electrochemical performance. The conductivity of the annealed samples increases by an order at high frequencies with the next saturation. With the increase of the annealing temperature, the equilibrium conductivity characteristic value decreases and its saturation is achieved at relatively lower frequencies. According to Dyre and Schrøder [18], the dependences *σ*(*ω*) of such type are characteristic for the case of materials disordered at the microscopic level.

**Fig. 5:**
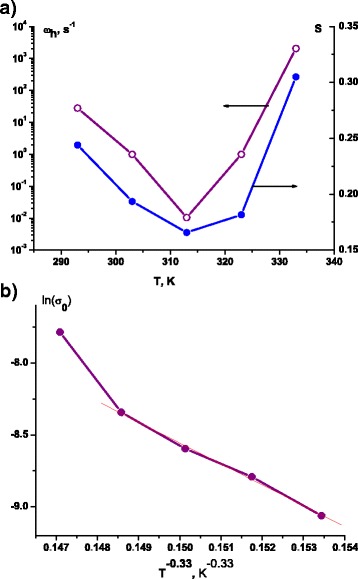
Temperature dependence of *ω*_h_ and *s* parameters (**a**) and ln(*σ*_0_) from (*T* ^− 0.33^) (**b**), characterizing the properties of the as-prepared nanocomposite MoS_2_/C with spherical particles

All the experimentally obtained *σ*(*ω*) curves were approximated by Jonscher’s power law in a form as (1) [19–21]

1 $$ \sigma \left(\omega \right)={\sigma}_{\mathrm{dc}}\left[1+{\left(\frac{\omega }{\omega_{\mathrm{h}}}\right)}^s\right], $$

where *σ*_dc_ is a conductivity at constant current; *ω*_h_ is a charge carrier hopping frequency; and *s* is an exponent, which characterizes the system deflection of the properties provided by Debye model, and is a measure of interparticle interactions, 0 < *s* < 1. From the received data, we have plotted the temperature dependences of *σ*_dc_, *ω*_h_ and *s*. It is found that in the case of as-prepared nanocomposite, MoS_2_/C parameter *s* varies nonlinearly with increasing temperature, getting the minimum value at 313 K and determining the change of carrier hopping frequency (Fig. 5a). The observed temperature dependence of *s* parameter even for such a narrow range of *T* change indicates the conduction mechanisms other than quantum tunneling of electrons and is a typical for good crystalline samples of MoS_2_ [22]. Apparently, the observed features are the result of the carbon presence. Probably, the temperature in the vicinity of 313 K activates the electron transition from the impurity levels in the forbidden zone. The results allowed to build the Arrhenius plot and determine the activation energy for the conduction of the material in the given temperature range, which is evaluated as 0.192 ± 0.010 еV and is in good agreement with published data for МоS_2_. Conductivity of bulk MoS_2_ is typically nonlinear dependent on temperature, in particular, the conductivity activation energy at the temperatures lower than room temperature lies in the range of 0.03–0.15 eV and above 760 K—0.4–0.9 еV [23]. Usually, such behavior is a characteristic of doped semiconductors with impurity conductivity below and intrinsic above 773 K.

Taking into account the system dimension, we have plotted ln(*σ*_0_) from (*T*^− 0.33^) (Fig. 5b), using Eq. 2

2 $$ \sigma (T)={\sigma}_0(T) \exp \left[-{\left(\frac{T_0}{T}\right)}^p\right], $$

where *p* = 1/(*d* + 1), and *d* is the dimensionality of the system; thus, in our case, *p* = 1/4 [24]. The resulting calculated value of the characteristic temperature *T*_0_ is 3.05 × 10^6^ K, which is several times higher than for atomically thin layers of MoS_2_ [25] and defines the effective density of the states near the Fermi level of the material, herewith the observed inverse dependence of these parameters.

In the case of materials obtained after annealing at 773 K in a stream of argon, the *σ*(*ω*) dependences (Fig. 4b) were approximated just partially to the zone of inflection. It was found that parameter *s* for this material is weakly dependent on the annealing temperature, varying within the approximation error within 0.33–0.37. Thus, we can assume that in this case, we observe the displays of quantum mechanical tunneling of charge carriers [23]. As it was already mentioned, the curves *σ*(*ω*) have a distinctive look, indicating a higher level of disorder. In this case, it becomes possible to use the symmetric hopping model (or random barrier model), whereby the charge transfer is a jump between close equilibrium positions in non-periodic potential [19]. In this model, the probability of hopping between individual positions is considered as the same with the normal distribution of potential barrier height, which provides no explicit value of activation energy. Reducing the equilibrium conductivity with increasing frequency and temperature is explained as follows: energy comes into the system as a result of both thermal excitation and application of the external periodic potential. At lower temperatures, the hops occur at higher frequencies, and thermal excitation effect is small. Thus, the frequency growth causes an increase of hop probability over the barriers, the heights of whose are distributed by the Gauss function, which explains the smooth curve growth progress—a sharp increase (area corresponded to the vicinity of mode value of the barrier height)—a saturation. With increasing temperature, this situation persists on providing the probability growth of carrier scattering on phonons and saturation at relatively lower frequencies.

**Fig. 6:**
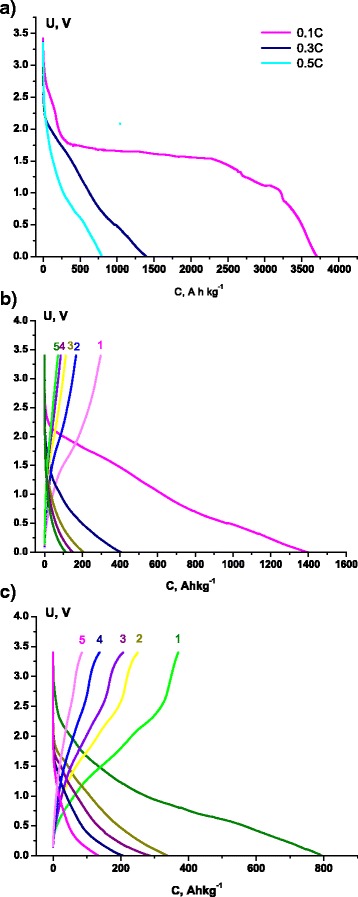
Charge/discharge curves at currents 0.1 (**a**), 0.3 (**b**), and 0.5 C (**c**)

For next study of electrochemical properties, we decided to take the MoS_2_/C multilayer nanospheres after thermal treatment, based on results described above. Galvanostatic measurements for this material as an electrode base for lithium power sources at currents 0.1, 0.3, and 0.5 C gave the specific capacity values at 3700, 1390, and 790 A h kg^−1^, respectively (Fig. 6). The calculated specific energy values are 5380, 1370, and 702 W h kg^−1^. Coulumbic efficiencies for the first and fifth discharge-charge cycles are 21.3 and 63.1 % at 0.3 C (Fig. 6b) and 46.6 and 63.4 % at 0.5 C (Fig. 6c). Such high values of specific capacity were not achieved before for any MoS_2_-based system presented in the literature.

**Fig. 7:**
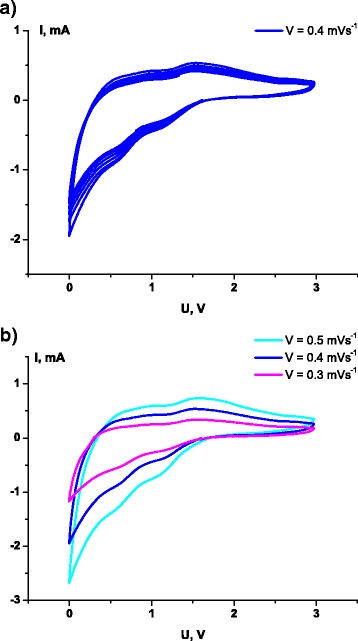
Cyclic voltammograms at different scan rates (**a**, **b**)

In turn, cyclic voltammograms (Fig. 7) in the 0–3.0 V potential window at different scanning rates (0.5, 0.4, and 0.3 mV s^−1^) correspond to quasi-reversible system and are very similar to CVs obtained for single-layered ultrasmall nanoplates MoS_2_ embedded in carbon nanowires [26]. Two cathodic peaks in the vicinity of 1 and 0.6 V can be attributed to the intercalation of Li^+^ into the interlayer spacing of MoS_2_ to form Li_x_MoS_2_, accompanied by phase transformation from the 2H (trigonal prismatic) to 1T (octahedral molybdenum coordination) MoS_2_ structure [27–29]. Electron transfer from lithium during intercalation changed the electron density causing the deformation of crystal structure. A peak at ~0.6 V is attributed to the following conversion reaction (3):

3 $$ {\mathrm{Li}}_{\mathrm{x}}{\mathrm{MoS}}_2+\left(2-x\right){\mathrm{Li}}^{+}+\left(2-x\right){e}^{-}\to {\mathrm{Li}}_2\mathrm{S}+\mathrm{M}\mathrm{o}, $$

anodic peak at approximately 1.7 V is due to the removal of Li^+^ ions and incomplete oxidation of Mo. The peak 2.3 V, which in the literature data corresponds to the formation of MoS_2_, is absent, signifying on the partial reversibility of the processes that occurred in the studied system. Moreover, Wang et al. [7, 27] assumed that Li_2_S is likely to be oxidized into S during the anodic scans. Thus, the possible reaction describes the Li storage mechanism as

4 $$ \mathrm{S} + 2\mathrm{L}\mathrm{i}\leftrightarrow {\mathrm{Li}}_2\mathrm{S}. $$

**Fig. 8:**
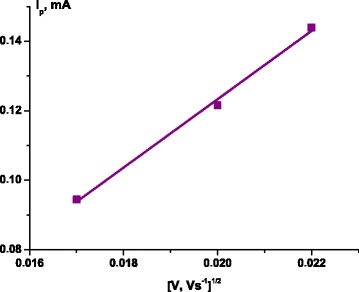
The dependence of current peaks on the square root of scanning rates calculated from cyclic voltammograms

The Randles-Ševcik Eq. (5) for such quasi-reversible system at 298 K defines the diffusion coefficient *D* as a slope of linear dependence of peak current *I*_p_ on the square root of scan rate *V* (Fig. 8) [30]

5 $$ {I}_{\mathrm{p}}^{\mathrm{quasi}}=2.65\times {10}^5{n}^{3/2}{\mathrm{ACD}}^{1/2}{\mathrm{V}}^{1/2}, $$

where *n* is the electron transfer number in the electrode reaction, *A* is the cathode surface, and *C* is the bulk concentration of electroactive particles. It is calculated in such manner that *D* = 1.65 × 10^−10^ cm^2^ s^−1^; however, it should be noted that the intercept of the resulted line does not equal zero, which signifies the complex rate-limiting processes, i.e., a mixture of diffusion and surface processes [31]. The studied redox species are partially adsorbed on the electrode surface or confined in a film matrix. This assumption could also explain the overall cyclic voltammograms look: the Li^+^ accumulation at the interfaces results in broadening and nearly rectangular shape between 0 and 1.4 V, which is a characteristic of supercapacitors [27]. According to [14], the intercalation and the pseudocapacity contributions can coexist. Thus, the high capacity value of MoS_2_/C multilayered nanospheres is a result of faradaic charge transfer processes and non-faradaic charge species storage at the interfaces or possibly at the inner surfaces of hollow multilayered nanospheres. The annealing caused numerous breaches, increasing the edges sites that have higher Li binding energies than the inner sites, implying a remarkable edge effect [9, 32, 33]. In particular, the S edge of the MoS_2_ is more favorable to bind Li than the Mo edge and favors the active material to achieve a high specific capacity due to the increasing number of intercalated Li atoms. In addition, the hollow spherical structure could effectively tolerate the volume change caused by the discharge-charge processes, reduce the diffusion distance of lithium ions, and facilitate the charge diffusion due to carbon presence. But still, there is not good cycling performance and capacity loss due to the electrolyte decomposition and inevitable formation of solid electrolyte interphase (SEI) and/or some lithium trapping inside the lattice [7].

## Conclusions

Spherical nanoparticles with alternating MoS_2_ and C layers synthesized by hydrothermal method were studied as an electrode base for Li power sources. It was determined that the obtained values of specific capacity (3700, 1390, and 790 A h kg^−1^ at currents 0.1, 0.3, and 0.5 C, respectively) are caused by synergetic effect of the following factors: (i) deformation, expanding, and breaches of MoS_2_ crystal structure as a result of carbon layers’ presence and thermal treatment; (ii) conductivity growth for MoS_2_/C nanocomposite comparatively to bulk materials; and (iii) combination both faradaic and pseudocapacitive non-faradaic mechanisms of charge accumulation. The conductivity character of the obtained MoS_2_/C composite is being changed after thermal treatment from typical for crystalline MoS_2_ to symmetric hopping or random barrier model. The conductivity saturation point, observed in the annealed material, is balancing between temperature and frequency of applied field and decreasing at higher temperatures. Without modifying the 2H structure of MoS_2_, the annealing has introduced a number of defects—the supplementary active sites—where the redox reactions occur. This together with spherical hollow structure of MoS_2_/C nanoparticles affected the results of galvanostatic and potentiodynamic studies.
